# The Impact of Biophysical Properties of Erythrocytes on their Aggregation

**Published:** 2017-06

**Authors:** Mohamed A. Elblbesy, Maisa E. Moustafa

**Affiliations:** 1 Department of Medical Biophysics, Medical Research Institute, Alexandria University, Egypt;; 2 Department of Medical Laboratory Technology, Faculty of Applied Medical Science, University of Tabuk, Saudi Arabia

**Keywords:** Erythrocytes, Aggregation, Mean cell volume, osmotic fragility, electrophoretic mobility, magnetophoretic mobility

## Abstract

Erythrocytes aggregation takes places under low shear conditions or at stasis. All suggested mechanisms of erythrocytes aggregation indicated the importance role of fibrinogen and other blood proteins in enhanced erythrocyte aggregation. Recently a special attention is given to the cellular factors that may effect on erythrocytes aggregation. The present study inferred the effect of the cellular properties of erythrocytes on their aggregation. In the present study, aggregation index was calculated by a simple microscopic method. Correlations between erythrocytes aggregation index and mean cell volume, osmotic fragility, electrophoretic mobility, and magnetophoretic mobility were studied. The findings of this study indicated that the aggregation index is significatly correlated to mean cell volume, magnetophoretic mobility, osmotic fragility and electrophoretic mobility. Thus, It is concluded that cellular factors should be taken into consideration when studying the mechanism of erythrocytes aggregation.

## INTRODUCTION

Erythrocyte aggregation is one of the major determinants of in vivo blood flow and blood viscosity at low shear rates. During the aggregation process, erythrocytes reversibly form rouleaux or network of aggregates (1).

Two mechanisms were suggested to explain the erythrocytes aggregation, protein bridging or by protein depletion mechanisms (2-4) . The bridging hypothesis guarantees that erythrocyte aggregation takes place when disaggregating forces are not competent to resist the adsorption of macromolecules to the cell surface, while the depletion mechanism poposes that the aggregates begin to form as protein concentration in the surrounding media (of erythrocytes) would decrease by leading to a osmotic gradient between to adjacent erythrocytes by a depletion interaction (5, 6). The precise mechanisms of erythrocytes aggregation has been yet unreadable. Although increased erythrocytes aggregation has been observed in various diseases (e.g., hypertension, diabetes mellitus), all mechanisms of the process and the relations between different pathological states and erythrocytes aggregation have not been entirely identified yet (7-9).

The effect of the viscoelastic properties of erythrocyte membranes, erythrocyte deformability, interactions between macromolecules and erythrocytes as well as hematocrit on erythrocytes aggregation is well documented (10, 11) . The earlier investigations of erythrocytes aggregation has focused fundamentally on the effects of properties the suspending media such as concentration and molecular size of dissolved proteins and macromolecules. It has been shown that erythrocytes characterizations are essential determinants of erythrocytes aggregation (12, 13). It has been demonstrated that cell modifications due to physiological or pathological conditions result in altered aggregation forms in both autologous plasma and standard aggregation media (14-17) . It has also been shown that erythrocytes aggregation varies widely among mammalian species and those differences persist when the erythrocytes are washed and re-suspended in standard aggregating media (18). The specific structural and functional basis for the differences mentioned above in erythrocyte aggregation tendency (i.e., erythrocyte “aggregability”) are not yet fully identified, although it is most likely that these differences reflect different erythrocyte surface properties, especially those involving the glycocalyx (19).

The majority of the previous studies focused on the role of external factors such as on fibrinogen concentration on erythrocytes aggregation. The present study concentrates on the effect of physical properties of erythrocytes on the aggregation process. Selected biophysical parameters; mean cell volume (MCV), osmotic fragility, electrophoretic mobility EPM, and magnetophoretic mobility (MPM) has been calculated for erythrocytes. Their correlations with aggregation index (AI) has been examined to estimate the role of biophysical parameters on the aggregation.

## MATERIALS AND METHODS

### Sample collection

Fifty blood samples were collected from healthy donors with the same age and gender. The stock was drawn after 10 minutes resting period and in a seated situation. The blood was taken in from the antecubital vein after 90 s from the application of tourniquet (20). EDTA with concentration1. 5 mg/ml was used as an anticoagulant. Two ml of each sample was sent to the clinical laboratory to measure mean corpuscle volume (MCV) and to make sure that all other indices of blood within the normal range.

### Erythrocytes aggregation measurement

Erythrocytes were resuspended in autologous plasma with a volume concentration of 1.0%. 200 µL of the suspension was admitted into a hemocytometer on a microscope stage. After two minutes the aggregation process was completed, and the erythrocytes aggregates became stationary. Images at 100 × magnification were taken from at least five different fields using CCD camera attached to the eyepiece of the microscope. The images transferred to the PC and analyzed using ImageJ software. Particles count tool was used to count cell units in each field, according to the definition that one “unit” was either one monodispersed cell or one aggregate. The same procedure was used to count cell units for erythrocytes suspended in PBS at the same concentration of 1.0%. Aggregation index (MAI) was calculated as the following:

eq. 1 $$\text{MAI}=\frac{\text{Cells units suspended in plasma}}{\text{Cells units suspended in PBS}}$$

### Osmotic Fragility

A set of PBS solutions ranging from 0.90% to 0.35% was prepared in different tubes. 0.01 ml of erythrocytes were added on 10 ml of PBS sample in each tube. Erythrocytes were mixed and incubated in PBS for 30 minutes at 4°C, followed by centrifugation for 12 minutes at 1100 × g. The free Hb in the supernatant was spectrophotometericaly determined. The concentration of PBS necessary to induce 50% hemolysis defined the osmotic fragility index of the erythrocytes.

### Electrophoretic mobility measurement

The speed of erythrocytes in an electrical field was determined in suspension using a microscopic technique. Standard microscope slides with the electrodes system consisted of two isolated gold coated bars with a separation distance of 1 mm placed directly on the glass slide. The electrodes were connected to power supply adjusted at 10V. Erythrocytes were suspended in 0.9% NaCl of pH 7.4 at a Hct of 0.05% and then were introduced between the electrodes. Inverted microscope attached to CCD was used to monitor the speed of erythrocytes inside the electric field. Electrophoretic mobility (EPM) was calculated as the following:

eq. 2 $$\text{EPM}=\frac{\text{s}}{\text{E}}$$

where s is the speed of erythrocytes (m/s), and E (V/m)the electric field strength.

### Magnetophoretic mobility measurement

Arrangement consists of coil for producing static magnetic field attached to glass slide and connected to power supply was built on the stage of an inverted microscope. Eyepiece camera was attached to the eyepiece of the microscope. For Magnetophoretic mobility (MM) measurement, erythrocytes at concentration 0.1% were introduce on the glass slide between the magnetic coil. Erythrocytes on the glass slide moved with a constant velocity perpendicular to the direction of magnetic field. The images of migrating erythrocytes were captured to determine the erythrocytes’ magnetic migration velocity (v) by dividing the migrated distance by time taken for the migration. MM for each erythrocyte was measured using the following equation:

eq. 3 $$\text{MM}=\frac{\text{v}}{\text{s}}$$

where s is magnetophoretic driving force on the cell.

## RESULTS AND DISCUSSION

**Figure 1:**
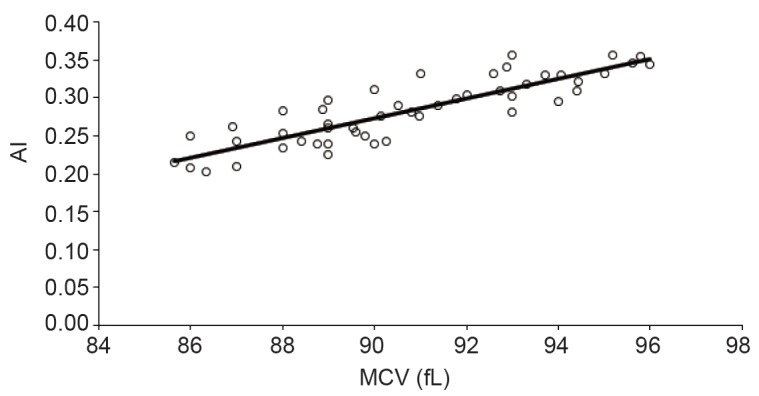
Correlation between aggregation index and mean cell volume.

The impact of the erythrocytes shape changes on sedimentation rate was monitored both in vivo and in vitro. The increase in sedimentation rate was observed in stomatocytosis. It was found that a severely abnormal red cell morphology reduced the sedimentation rate in a standardized, fibrinogen-rich plasma to about half. These results indicate that the shape plays a crucial role in the aggregation and sedimentation of erythrocytes and they may contribute to the understanding of the interaction of erythrocytes with other cells such as endothelium (21, 22) . Our results indicated positive correlation between AI and MCV (Figure 1) with strong correlation (*R* ^2^ > 0.6)  between them. The increasing in cell volume lead more flatting which increase the tendency of aggregation. Also, the large volume offer more contact area between erythrocytes which leads to more aggregation with lower chance of irreversible action.

**Figure 2:**
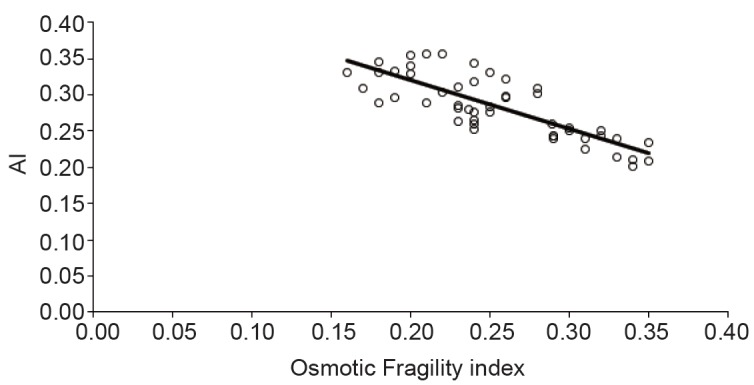
Correlation between aggregation index (AI) and osmotic fragility index.

Membrane flexibility of erythrocytes is an important determinant of the cell aggregation. Stiffening of the cell membrane results in decreased aggregation because cell-to-cell contact is impeded (23). It was indicated that as the surface area to cell volume ratio decreased at acidic pH cell-to-cell contact might be suppressed (24). Recently, it has been demonstrated that deformability of erythrocytes with constant volume at various pH levels (6.2–8.0), which was achieved by varying osmolarity, significantly decreased at lower pH because of the change in membrane elastic properties (25). Iwona Cicha *et al*. studied the erythrocytes aggregation at pH 6.5–8.7 and they identified a significant change in erythrocytes aggregation (26). To form erythrocyte rouleaux erythrocytes must change their shape hence deformability is an important cellular property for aggregation. The properties of the glycocalyx on the exterior of the erythrocyte membrane is also important determinants of aggregation behavior (27-29). Figure 2 showed strong negative correlation between MAI and osmotic fragility of erythrocytes. Osmotic fragility reflects the elasticity of the cell membrane. The dependency of the cells aggregation on the cell elasticity was proved as mentioned above. Our results confirmed the role of the cell membrane elasticity in aggregation.

**Figure 3:**
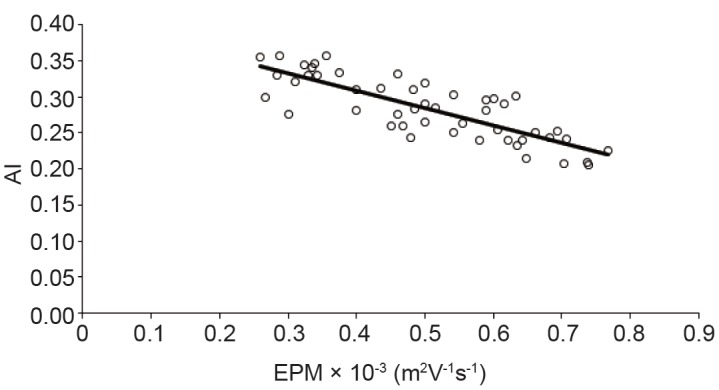
Correlation between aggregation index (AI) and electrophoretic mobility (EPM).

It is known that erythrocytes membrane determine their physiological functions. There is always a separation distance between erythrocytes. This separation is mainly determined by the electrostatic repulsion of negatively surface charges. Electrophoretic mobility of erythrocytes is the most useful parameter for estimating surface charge of erythrocyte (30). Electrophoretic mobility plays an important rule in the Bridging Model, and Depletion Model for erythrocytes aggregation include either direct or indirect aspects of the erythrocyte. Many studies examined the relation between the aggregation and EPM (12, 31-34) . It was mentioned that the surface charge of erythrocytes or binding of fibrinogen to the cells are some important factors affecting erythrocytes aggregation (35, 36). Oguz K. Baskurt *et al*. explored the inverse relation between rats, human or horse erythrocytes aggregation in plasma and EPM in buffer with (p > 0.1) (37). I. A. Tikhomirova *et al.* demonstrated that as the electrophoretic mobility did not reduce the aggregability strength of erythrocytes. Theoretically, increasing in the surface charge of the erythrocytes reduce their aggregation due to increasing in electrostatic repulsion. In contradictory of this theoretical assumption, experimental data showed a positive correlation between the negative electrostatic charge of the membranes and the aggregability (38). Polikar indicated that not only the electrostatic force paly the major role in cell to cell interaction but other opposite forces such as van der Waals attraction and some chemical interactions should be taken into account. Although the existence of a mutual influence of contacting cells is well known, its mechanisms have not been studied in detail (39). Some studies indicated that the minor effect of electrostatic force on erythrocytes aggregation (40). Our results (Figure 3) indicated a strong negative correlation between AMI and EPM of the erythrocytes (*R* ^2^ > 0.6). This is in compatible with some of the previous studies. Reduction in the erythrocytes aggregation may be due to a change electrical repulsive forces of erythrocytes membrane.

**Figure 4:**
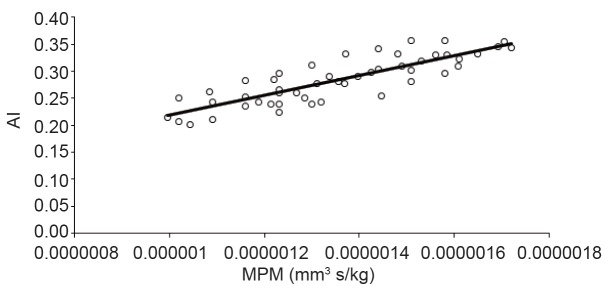
Correlation between aggregation index (AI) and magnetophoretic mobility (MPM).

Hemoglobin is one of the main protein of erythrocytes and it is capable of binding oxygen molecules due to its iron content. The hemoglobin's molecular structure configuration of strongly depends on the presence of oxygen. The oxygenated hemoglobin is called oxyhemoglobin, otherwise, is called deoxyhemoglobin (41). Erythrocytes can be considered as small magnetic particles. The erythrocytes can be either paramagnetic or diamagnetic, depending on their hemoglobin oxygenation (42). Oxygen saturation of Hb is directly associated with its magnetic property. The oxygenated erythrocyte is diamagnetic (due to absence unpaired electrons) when compared with deoxygenated erythrocyte which is paramagnetic in nature (due to the presence of unpaired electrons in Fe_2_ of their heme moieties). Due to the difference in magnetic susceptibility, the magnetophoretic mobility of the oxygenated and deoxygenated erythrocytes would change (43). Tao, R, *et al*. reported that blood viscosity could be reduced with magnetic fields of 1 T or above in the blood flow direction. Also, they indicated that without a magnetic field applied, randomly distribution of erythrocytes was observed in the plasma. After gradually increasing to a strong magnetic field level (1.3 T) for 12 min the erythrocytes aggregated to form long cluster chains. (44). Uyuklu, M. *et al*. indicated that erythrocytes aggregation parameters were affected by oxygenation if measured based on light backscattering (45). Based on the previous investigations aggregation is strongly dependent on hemoglobin concentration and also in the oxygenated state of the hemoglobin. Magnetic properties of erythrocytes as well as aggregation dependents on hemoglobin content and structure. Figure 4 showed the dependency of erythrocytes aggregation on their magnetic properties. Strong correlation between AI and MPM was observed (*R* ^2^ > 0.6).

## CONCLUSION

Erythrocytes aggregation is a very complex process that effected by many factors. The cellular factors are crucial as well as the plasma and blood flow factors. The biophysical properties of the erythrocytes have a significant effect on the aggregation process. Thus, they should be taken into consideration when investgating the mechanisms of erythrocytes aggregation.
